# ERBB2-CAR-Engineered Cytokine-Induced Killer Cells Exhibit Both CAR-Mediated and Innate Immunity Against High-Risk Rhabdomyosarcoma

**DOI:** 10.3389/fimmu.2020.581468

**Published:** 2020-10-19

**Authors:** Michael Merker, Juliane Wagner, Hermann Kreyenberg, Catrin Heim, Laura M. Moser, Winfried S. Wels, Halvard Bonig, Zoltán Ivics, Evelyn Ullrich, Thomas Klingebiel, Peter Bader, Eva Rettinger

**Affiliations:** ^1^Division for Stem Cell Transplantation, Immunology, and Intensive Care Medicine, Department for Children and Adolescents, University Hospital Frankfurt, Goethe University Frankfurt, Frankfurt, Germany; ^2^German Cancer Consortium (DKTK), Partner Site Frankfurt/Mainz, Frankfurt, Germany; ^3^Frankfurt Cancer Institute, Goethe University, Frankfurt, Germany; ^4^Division of Medical Biotechnology, Paul-Ehrlich-Institute, Federal Institute for Vaccines and Biomedicines, Langen, Germany; ^5^Georg-Speyer-Haus, Institute for Tumor Biology and Experimental Therapy, Frankfurt, Germany; ^6^Department of Cellular Therapeutics/Cell Processing (Good Manufacturing Practice, GMP), Institute for Transfusion Medicine and Immunotherapy, Goethe University, Frankfurt, Germany; ^7^Division of Hematology, Department of Medicine, University of Washington, Seattle, WA, United States; ^8^Experimental Immunology, Department for Children and Adolescents, University Hospital Frankfurt, Goethe University, Frankfurt, Germany

**Keywords:** cellular therapy, cytokine-induced killer cells, chimeric antigen receptor, rhabdomyosarcoma, ERBB2 (HER2/neu)

## Abstract

High-risk rhabdomyosarcoma (RMS) occurring in childhood to young adulthood is associated with a poor prognosis; especially children above the age of 10 with advanced stage alveolar RMS still succumb to the disease within a median of 2 years. The advent of chimeric antigen receptor (CAR)-engineered T cells marked significant progress in the treatment of refractory B cell malignancies, but experience for solid tumors has proven challenging. We speculate that this is at least in part due to the poor quality of the patient's own T cells and therefore propose using CAR-modified cytokine-induced killer (CIK) cells as effector cells. CIK cells are a heterogeneous population of polyclonal T cells that acquire phenotypic and cytotoxic properties of natural killer (NK) cells through the cultivation process, becoming so-called T-NK cells. CIK cells can be genetically modified to express CARs. They are minimally alloreactive and can therefore be acquired from haploidentical first-degree relatives. Here, we explored the potential of ERBB2-CAR-modified random-donor CIK cells as a treatment for RMS in xenotolerant mice bearing disseminated high-risk RMS tumors. In otherwise untreated mice, RMS tumors engrafted 13–35 days after intravenous tumor cell injection, as shown by *in vivo* bioluminescence imaging, immunohistochemistry, and polymerase chain reaction for human gDNA, and mice died shortly thereafter (median/range: 62/56–66 days, *n* = 5). Wild-type (WT) CIK cells given at an early stage delayed and eliminated RMS engraftment in 4 of 6 (67%) mice, while ERBB2-CAR CIK cells inhibited initial tumor load in 8 of 8 (100%) mice. WT CIK cells were detectable but not as active as CAR CIK cells at distant tumor sites. CIK cell therapies during advanced RMS delayed but did not inhibit tumor progression compared to untreated controls. ERBB2-CAR CIK cell therapy also supported innate immunity as evidenced by selective accumulation of NK and T-NK cell subpopulations in disseminated RMS tumors, which was not observed for WT CIK cells. Our data underscore the power of heterogenous immune cell populations (T, NK, and T-NK cells) to control solid tumors, which can be further enhanced with CARs, suggesting ERBB2-CAR CIK cells as a potential treatment for high-risk RMS.

## Introduction

The immune system recognizes and destroys tumor cells through a process known as immunosurveillance. However, especially in advanced disease, tumors escape immunosurveillance by cancer immunoediting and an immunosuppressive tumor microenvironment (TME). Despite improvements in surgical and radiotherapy techniques, new chemotherapy regimens, and the use of allogeneic stem cell transplantation, children, and young adults with metastatic alveolar rhabdomyosarcoma (RMS)—except those younger than 10 years of age—still succumb to their disease within a median of 2 years (1–8). Thus, all treatment advances made over the last three decades have not translated into improved outcomes in high-risk RMS patients.

In recent years, targeted immunotherapies have emerged as a therapeutic strategy that interferes with cancer cell growth and spread or triggers antitumor immunity. By directly transferring cell products with specific antitumor properties, innate and adoptive immune responses against tumors and tumor-associated antigens (TAAs) can be triggered or enhanced. In this context, adoptive cell therapy (ACT) with chimeric antigen receptor (CAR)-modified patient immune cells is attracting growing interest.

In this fast-moving field, a growing number of CAR-engineered cell products have emerged, although most involve autologous T cells targeting hematopoietic malignancies. Only a few approaches are used in targeting solid cancer (9–18). As surface expression of ERBB2 is detectable in a substantial subset of alveolar RMS and other tumor entities (19, 20), the use of CAR T cells targeting ERBB2 was developed in the context of ACT for soft tissue sarcoma (STS). This treatment was found to be safe in a phase I/II clinical trial, but induction of long-lasting immune responses was only possible in a minority of patients (9, 21, 22). We expect that this is at least in part due to the poor quality of autologous T cells after chemotherapy pretreatment and therefore propose using CAR-modified cytokine-induced killer (CIK) cells derived from healthy donors or patients' own apheresis prior to chemotherapy treatment.

CIK cells, which are generated from peripheral blood mononuclear cells (PBMC) in the presence of defined cytokines *in vitro*, are a heterogeneous cell population characterized by CD3^+^ T cells with a CD56^+^ natural killer (NK) cell phenotype, a high proliferative rate *in vitro*, and strong lytic activity against a broad spectrum of cancers (23, 24). CIK cell cytotoxicity is mostly attributed to the CD3^+^CD56^+^ T-NK cell fraction. T-NK cells are terminally differentiated non-proliferating cells derived from proliferating progenitor T cells in *in vitro* cultures. Pievani et al. reported that T-NK cells have a dual functional capability by preserving T cell receptor (TCR)-mediated specific cytotoxicity and acquiring non-major histocompatibility complex (MHC) restricted, inherently broader NK cell function (25). The NK cell-like cytotoxic capacity of CIK cells mediated *via* several receptors, such as NKp30, DNAM-1, and LFA-1, has mainly been ascribed to NKG2D, an activating NK cell receptor. The first reports by Schmidt-Wolf et al. documented the efficacy and safety of CIK cell treatment in different cancers (23, 26, 27). Since then, a wide variety of phase I/II clinical trials recorded in the International Registry on CIK cells (IRCC) have shown that adjuvant CIK cell therapy with or without chemotherapy or other therapeutic regimens, may prevent disease recurrence, improve progression-free and overall survival, and enhance the quality of life of cancer patients with only minimal and manageable toxicity and side effects (28–30).

We previously showed that CIK cells, which are already capable of NK cell-like antitumor function, can be supplemented with an ERBB2-CAR construct that provided synergistic activities *in vitro* (31). The alveolar RMS cell line RH30 which was established from the bone marrow (BM) metastasis of a 17-year-old male patient was used for preclinical *in vivo* analysis. Here we present an ACT approach targeting CIK cells to ERBB2 with a second-generation CAR for the treatment of primarily disseminated high-risk alveolar RMS in a complete new xenograft model.

## Materials and Methods

### Generation of Wild-Type (WT) CIK Cells

WT IL-15-activated CIK cells were generated from the PBMCs of healthy volunteers after written informed consent and the study was approved by the Ethics Review Board of the Medical Faculty of the University Hospital Frankfurt/Main, Germany (Geschäfts-Nr. 413/15).

CIK cells were generated from PBMCs after standard Ficoll separation as previously described (32). In brief, cells were resuspended at 3 × 10^6^ cells/mL in RPMI 1640 medium supplemented with 10% FCS, L-glutamine, antibiotics and 1,000 U/mL IFN-γ. On day 1 of culture, 100 ng/mL anti-CD3 antibody (MACS GMP CD3 pure, Miltenyi Biotech, Bergisch Gladbach, Germany) and 500 U/mL IL-2 were added. Starting at day 3 of culture, cells were resuspended at 1 × 10^6^ cells/mL and expanded in the presence of 50 ng/mL IL-15 (PeproTech, Hamburg, Germany). On day 4 to day 7 of culture, WT and ERBB2-CAR-engineered CIK cells (described below), were both cultured at ~5 × 10^5^ cells/2 mL in 6-well plates. On day 7 of culture, cell products were again transferred to culture flasks, resuspended at 1 × 10^6^ cells/mL and supplemented with 50 ng/mL IL-15 every 3 days. On day 12 of culture, cell products were harvested and used for *in vitro* and *in vivo* analysis.

### CAR Engineering Using the ERBB2-Specific Lentiviral CAR Vector pS-5.28.z-IEW

The lentiviral CAR vector pS-5.28.z-IEW, which encodes an ERBB2-specific second-generation CAR, was described previously (33). The codon-optimized CAR sequence consists of an IgG heavy-chain signal peptide, an ERBB2-specific scFv antibody fragment (FRP5), and a modified CD8α hinge region, as well as CD28 transmembrane and intracellular domains and a CD3ζ intracellular domain (CAR 5.28.z), and was inserted into a pHR'SIN-cPPT-SIEW (pSIEW) (34) lentiviral transfer plasmid upstream of the IRES and enhanced green fluorescent protein (eGFP) sequences. eGFP was used as a fluorescent marker. Vesicular stomatitis virus G (VSV-G) protein pseudotyped lentiviral vector particles were produced using the lentiviral transfer plasmid with the packaging and envelope plasmids pCMVΔR8.91 and pMD2.G as described previously (35).

Transduction of CIK cells with lentiviral vector-containing supernatant was carried out at day 4 of the expansion culture, 24 h after the first stimulation with IL-15, as described previously (31). The culture was then adjusted to 1 × 10^6^ cells/mL every 3–4 days and supplemented with 50 ng/mL IL-15 (described above). On day 12 of culture, the cells were harvested and used for *in vitro* and *in vivo* analysis.

### Generation of a Luciferase-Expressing RH30 Cell Line

The alveolar RMS cell line RH30 was purchased from DSMZ (Deutsche Sammlung von Mikroorganismen und Zellkulturen GmbH, Braunschweig, Germany) and cultured according to the manufacturer's instructions. The RH30 cell line, which was established from the BM metastasis of a heavily pretreated 17-year-old male patient with refractory alveolar RMS (p53 mutation- and Pax3/FKHR fusion protein-positive), was selected in regard to clinical translatability (36–38).

To image the *in vivo* trafficking of tumor cells, GFP/luciferase-expressing RH30 cells (RH30^GFP/Luc^) were generated *via* lentiviral transduction using vector particles pseudotyped with the VSV-G protein that were produced using the transfer plasmid pSEW-luc2, which encodes firefly luciferase and eGFP linked *via* a 2A peptide (39). GFP-positive cells were enriched by fluorescence-activated cell sorting (FACS) using a FACSAria II™ instrument (BD Biosciences, San Jose, CA, USA).

### Preclinical Human RMS Mouse Model

NOD/SCID/*IL-2R*γ*c*^−/−^ (NSG) mice were purchased from The Jackson Laboratory (Bar Harbor, ME, USA) and maintained in the animal facilities of Georg-Speyer-Haus, Institute for Tumor Biology and Experimental Therapy, Frankfurt/Main, Germany. The described research was approved by the appropriate government committee (Regierungspräsidium Darmstadt, Germany; Gen.-Nr. TVA FK/1000) and conducted in accordance with the requirements of the German Animal Welfare Act.

**Figure 1:**
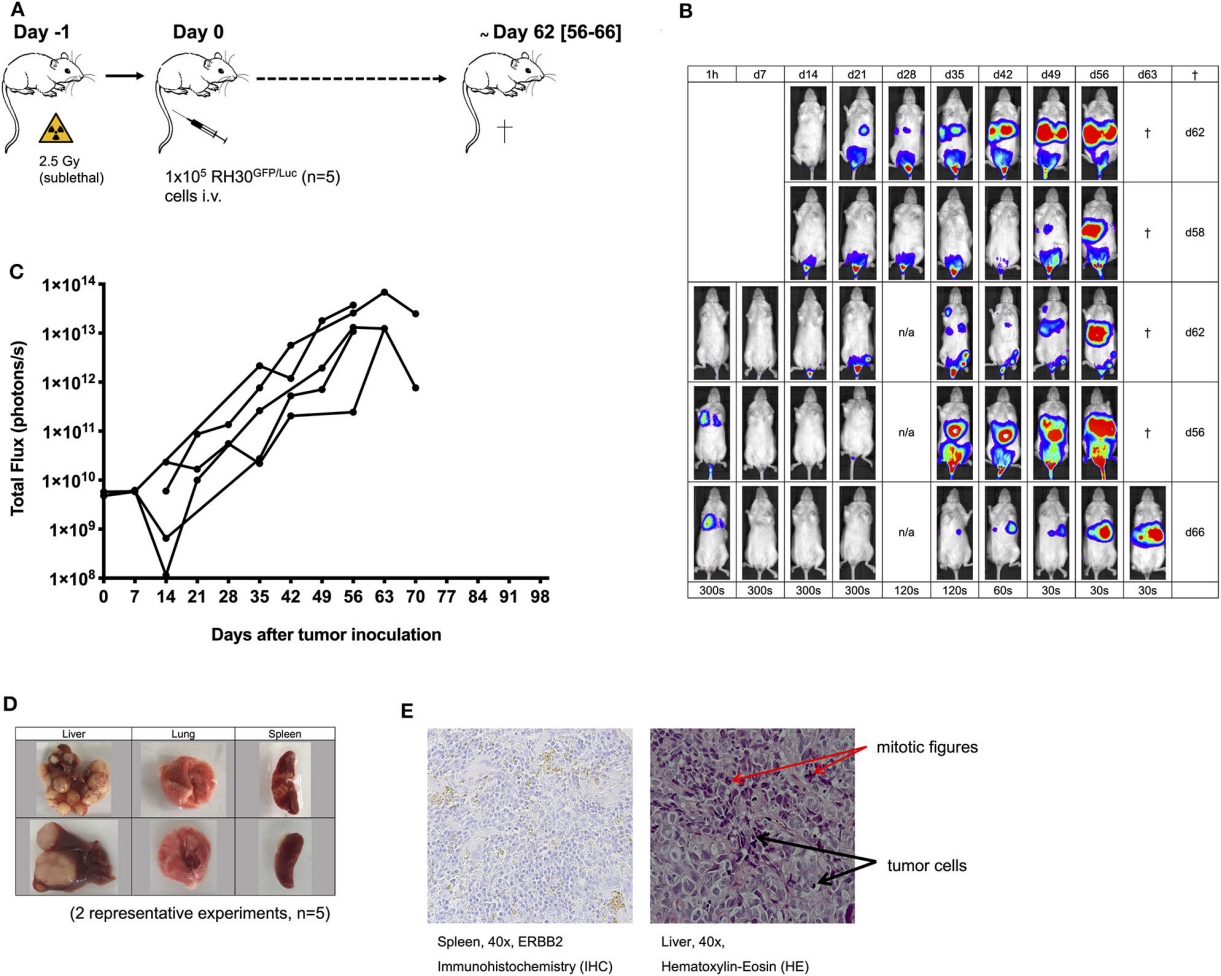
Establishment of the human RH30^GFP/Luc^ mouse tumor model. The establishment of a human luciferase-expressing RH30^GFP/Luc^ cell line **(A)** was monitored by bioluminescence imaging (BLI; **B,C**). Tumor engraftment was confirmed by macroscopic examination (**D**, two representative experiments shown), histology [hematoxylin-eosin (HE) staining; **E**], immunohistochemistry (IHC; staining for human ERBB2; **E**), and PCR analysis (Figure 2F).

To establish a completely new disseminated human RMS model in mice best mimicking the clinical situation of residual circulating tumor cells and refractory tumors following chemo- and radiotherapy, 6- to 8-week-old female NSG mice were sublethally irradiated with 250 cGy (Biobeam 2000, Eckert & Ziegler, Bebig, Germany) 24 h (day −1) prior to intravenous injection of 1 × 10^5^ luciferase-expressing RH30^GFP/Luc^ cells applied in a total volume of 100 μL per mouse *via* the tail vein (day 0) (Figure 1A).

As this study includes a comparisons of mice without and with WT or ERBB2-CAR CIK cell therapies which were given preemptively (day +1 and day +36) to mice at risk for tumor progression as well as to mice with established tumors (day +22 and day +57), 28 mice were randomly divided into 5 different treatment groups:

- Control: Dulbecco's phosphate-buffered saline (DPBS) on day +1, *n* = 5
- WT, preemptive: 2.5 × 10^6^ WT CIK cells on day +1 and day +36 (5 weeks, if possible), *n* = 6
- WT, established tumor: 2.5 × 10^6^ WT CIK cells on day +22 (3 weeks) and day +57 (8 weeks, if possible), *n* = 3
- CAR-CIK, preemptive: 2.5 × 10^6^ ERBB2-CAR CIK cells on day +1 and day +36 (5 weeks, if possible), *n* = 8
- CAR-CIK, established tumor: 2.5 × 10^6^ ERBB2-CAR CIK cells on day +22 (3 weeks) and day +57 (8 weeks, if possible), *n* = 6

All mice treated preemptively with WT or ERBB2-CAR CIK cells had minimal residual, but already active disease at the time of immune cell infusions, thereby considering them as having an imminent risk for disease progression with limited treatment options.

During the course of the experiment, mice were screened for symptoms of disease and adverse side effects like xenogeneic graft-vs.-host disease (xGVHD) and cytokine-release-syndrom (CRS) at least twice daily for a maximum of 100 days. Mice showing visible signs of poor health or physical abnormalities were painlessly euthanized with carbon dioxide asphyxiation followed by cervical dislocation. All animals were sacrificed after a maximum of 100 days and tumors as well as potential tumor- and xGVHD-targeted organs were excised for further analysis.

### Bioluminescence Imaging

Tumor growth was monitored weekly by bioluminescence imaging (BLI) using an IVIS Lumina II *in vivo* imaging system (Perkin Elmer, Waltham, MA, USA). Mice were anesthetized by isoflurane inhalation and subcutaneously injected with 150 μg of *in vivo*-grade VivoGlo™ luciferin (Promega, Madison, WI, USA) dissolved in 100 μL of DPBS per mouse. Images were acquired after an incubation time of 15 min. Data were recorded and analyzed using Living Image *in vivo* Imaging Software (Perkin Elmer). Total flux (photons/s) was used for measurement and statistical analysis of the tumor burden using a uniform region of interest in all mice.

### Harvest of Human Cells From the Organs of NSG Mice

Peripheral blood (PB), BM, lung, liver, gut, and spleen samples were excised and analyzed for occurrence of tumor and immune effector cells at the end of experiments. For this purpose, cell suspensions were prepared from the PB, BM, lung, liver, and spleen. Briefly, BM cells were collected from each tibia and femur by flushing the bones with culture medium. Mouse erythrocytes within BM and PB samples were lysed with lysis buffer (Mouse Erythrocyte Lysing Kit, R&D Systems, Wiesbaden, Germany) and washed once with washing buffer according to the manufacturer's instructions. Cell suspensions prepared from mouse organs digested with collagenase were filtered through a 100-μM cell strainer and washed with PBS. Aliquots of cell suspensions were analyzed by flow cytometry and quantitative polymerase chain reaction (qPCR).

### Flow Cytometry

WT and ERBB2-CAR CIK cells were characterized by flow cytometry prior to intravenous injection and at the end of experiments, if applicable after being harvested from organs of mice. Cells were washed once in PBS, resuspended in 100 μL of PBS, and stained with fluorescein peridinin chlorophyll (PerCP)-conjugated anti-human CD3, phycoerythrin (PE)-conjugated anti-human CD4, phycoerythrin-cyanin 7 (PE/Cy7)-conjugated anti-human CD56, allophycocyanin (APC)-conjugated anti-human CD8 or CD45RO, and allophycocyanin-cyanin 7 (APC/Cy7)-conjugated anti-human CD45 or CD8 antibodies or pacific blue-conjugated anti-human CD62L antibody.

To detect the cell surface expression of ERBB2-CARs, we labeled the CIK cells with an ERBB2 fusion protein as the primary reagent (ERBB2-IgG-Fc chimera, Sino Biological Inc., Beijing, P.R. China) after unspecific Fc-receptor blocking using TruStain FcX (Fc-Receptor Blocking Solution, BioLegend, San Diego, CA, USA). A secondary anti-IgG- Fc monoclonal antibody conjugated with APC was used to detect the primary ERBB2-IgG-Fc chimera (31).

All the antibodies were obtained from BioLegend (San Diego, CA, USA) unless otherwise specified. Isotype-matched fluorochrome-conjugated IgGs were used as controls.

Gates were set on viable lymphocytes, and the data were used for further analysis if at least 4 x 10^4^ CD45^+^/CD3^+^ events were acquired using a BD FACSCanto II flow cytometer (BD Biosciences, San Jose, CA, USA) with FACSDiva software (Version 6.1.3, BD Biosciences). Analyses were performed using FlowJo X software (Version 10.6.2, Tree Star Inc., Ashland, OR, USA). All multicolor flow cytometry assays with two or more colors were adjusted for spectral overlap.

### Chimerism Analysis

Genomic DNA was extracted using a QIAamp blood and tissue kit (Qiagen, Hilden, Germany). As a first step, a quantitative real-time PCR approach was used to assess the number of human cells in each tissue sample by specific amplification of the human albumin gene (40, 41). For each reaction, 50 ng of DNA was processed. This assay could detect one human cell in 1,000 murine cells. As a second step—within the human cell fraction—the proportions of CIK (WT and ERBB2-CAR) and tumor (RH30) cells were discriminated by a human-specific STR genotyping approach, similar to chimerism analyses (42). The tumor burden of each mouse was determined by evaluating tumor-specific STR signals per organ. Primers and probes were obtained from Eurofins (Eurofins MWG GmbH, Ebersberg, Germany) and genotyping of cell lines was performed using the STR multiplex PCR system Powerplex 16 (Promega GmbH, Mannheim, Germany).

### Histology and Immunohistochemistry

Histopathology of the tumor- and xGVHD-targeted internal organs of mice injected with WT or ERBB2-CAR CIK cells was performed by an external laboratory (mfd diagnostics GmbH, Wendelsheim, Germany) to assess antitumor capacity and alloreactivity. Experimenters were blinded to the treatment the mice had received. Tissue was fixed in 4% buffered formalin, paraffin embedded, sectioned, and stained with hematoxylin-eosin (HE) or immunohistochemistry (IHC) antibodies targeting human CD3 or ERBB2. A Zeiss AXIO Imager A1/M1 was used for microscopic examination of tumors and immune cell infiltration, as well as evaluation of xGVHD criteria.

### Statistics

Immune effectors cells (WT and ERBB2-CAR CIK cells) unless otherwise published were analyzed for surface markers, CAR expression, anti-tumor ability, and proliferation *in vitro*.

Groups of mice without and with immune cell therapies—which were given to mice at risk for tumor progression as well as to mice with established tumors—were compared regarding disease occurrence and immune cell infiltration as well as for adverse side effects and xGVHD. Animals were observed for 100 days and sacrificed for further analyses at day +100 of the experiment.

Differences between groups were evaluated by one-way ANOVA using the Holm-Sidak or Bonferroni-Dunn (non-parametric) method. A *p* < 0.05 was considered significant. Statistical analyses were performed using GraphPad Prism software (version 8.4, GraphPad Software, La Jolla, CA). Results are presented as disease free survival curves until day +100 or mean values ± standard errors of the mean (figures) and mean values ± standard deviations (results).

## Results

### Establishment of a Human RMS Xenograft Model in Immunodeficient Mice

**Figure 2:**
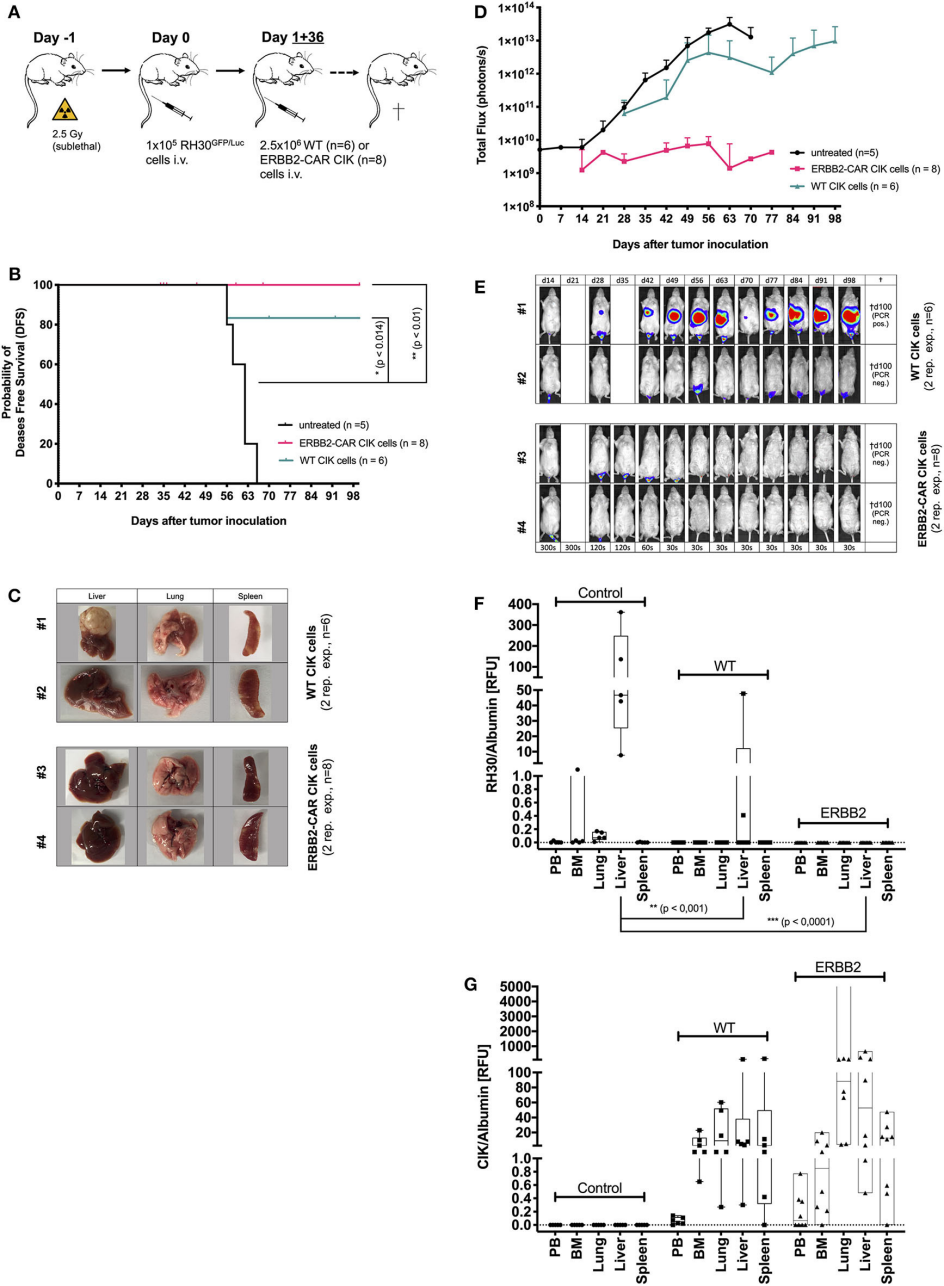
Cytotoxicity of WT and ERBB2-CAR CIK cells against minimal residual disease. The cytotoxicity of WT and ERBB2-CAR CIK cells against mice with minimal residual disease established with the human RH30^GFP/Luc^ cell line (preemptive treatment) was analyzed *in vivo* **(A)**. The disease-free survival **(B)** and tumor burden (evaluated by BLI) of WT and ERBB2-CAR CIK cell-treated mice (**D, E**; WT, *n* = 6 and ERBB2-CAR CIK, *n* = 8, respectively) were monitored. Tumor clearance was confirmed macroscopically (**C**, two representative experiments per group are shown) and tumor engraftment was analyzed by PCR **(F)**. CD3-positive human effector cells were identified in WT and ERBB2-CAR CIK cell-treated mice by immunohistochemistry (Table 1) and PCR **(G)**.

Establishment of alveolar human RMS xenografts using 1 × 10^5^ luciferase-expressing RH30^GFP/Luc^ cells (Figure 1A) was feasible in all mice (*n* = 5). Engraftment and organ distribution of RH30^GFP/Luc^ cells could be monitored by BLI between days +13 and +35 (Figure 1B) after tumor inoculation. Tumor engraftment was reliable, rapid, and life limiting at a median of 62 days after intravenous injection of tumor cells. The pattern of tumor growth was exponential and established in the BM, lung, liver, and spleen (Figures 1B–E). Mice had to be sacrificed because of visible signs of poor health (e.g., paralysis or visible tumor burden) between days +56 and +66 (median +62 days, Figures 1A,B). Macroscopic engraftment of human RMS (Figure 1D) was confirmed histologically by HE and IHC staining for human ERBB2 as well as by PCR (Figures 1E, 2F,G). High amounts of tumor cells were observed in the liver tissues of mice, whereas BM, lung, and spleen samples showed much lower human RMS engraftment. Human ERBB2 could be identified on tumor cells from spleens *via* IHC, whereas detection in intrahepatic cancer tissue (with more mitotic activity) was not possible due to autofluorescence of the liver tissue and bile.

### Generation, Expansion, and Characterization of the WT and ERBB2-CAR CIK Cells *in vitro*

WT and ERBB2-CAR CIK cells were generated from PBMCs of the same (single) healthy donor for immediate *in vivo* use after quality assurance (phenotypic characterization, proliferation, and viability). Applied batches of WT or ERBB2-CAR CIK cells did not differ in quality.

CIK cells were generated by stimulation with IFN-γ, IL-2, and anti-CD3 antibody, followed by addition of IL-15 starting at day 3 of the culture. On day 4, CIK cells were transduced with the lentiviral CAR vector. After incubation for 12 days, cell numbers of WT and ERBB2-CAR CIK cells increased up to 36.5-fold, SD ±23.4 and 13.4-fold, SD ±6.6.) with over >90% cell viability. Differences between WT and ERBB2-CAR CIK cells were not significant (*p* > 0.06). Furthermore, no difference in the proportions of CD3^+^CD56^−^ T cells (93.3% SD ±2.1 and 93.84% SD ±1.4; *p* > 0.95), CD3^+^CD56^+^ T-NK cells (4.8% SD ±2.0 and 4.1% SD ±1.4; *p* > 0.28), and CD3^−^CD56^+^ NK cells (0.71% SD ±0.18 and 0.79% SD ±0.26; *p* > 0.73) between WT and ERBB2-CAR CIK cells were found during a 12 day culture period. In contrast, the proportion of CD4^+^ (33.9% SD ±2.5 and 14.4% SD ±3.8; *p* < 0.009) and CD8^+^ (59.9% SD ±3.1 and 75.4 SD ±4.2; *p* < 0.03) subpopulations differed significantly between WT and ERBB2-CAR CIK cells (Supplementary Figure 1A). Interestingly, prior to infusion, ERBB2-CAR CIK cells displayed significantly more helper T cells with effector memory phenotype compared to WT CIK cells, whereas the latter contained more naïve T cells (Supplementary Figures 1C,D). Mean transduction efficiency determined *via* eGFP expression of transduced CIK cells was 21.1% (SD ±8.8%, *n* = 7), with 84.3% (SD ±6.9%, *n* = 3) of these cells also displaying high CAR expression on the cell surface (Supplementary Figures 1A,B).

### Preemptive WT and ERBB2-CAR CIK Cell Treatment in RMS Xenografts

After reliably and successfully establishing the xenograft RMS mouse model as a control, the potential of WT and ERBB2-CAR CIK cells to reach and eliminate inoculated ERBB2-positive tumors was investigated. At first, immune cell therapies were given preemptively (day +1 and day +36 after RH30^GFP/Luc^ tumor cell injection) (Figure 2A). All mice treated preemptively with WT or ERBB2-CAR CIK cells had minimal residual, but already active disease at the time of immune cell infusions, which translated into an imminent risk for disease progression with limited treatment options in a clinical setting. Hence, all mice without immune cell therapies showed progressive tumor growth and died within 56–66 days. Remaining animals were sacrificed at day +100 of the experiment and organs were excised for further analysis. Preemptive WT and ERBB2-CAR CIK cell treatment significantly improved disease-free survival until day +100 when compared to PBS-treated controls (*p* < 0.014 and *p* < 0.01), respectively, without differences between WT and ERBB2-CAR CIK cells (Figure 2B). In addition, BLI confirmed sustained inhibition of tumor engraftment in all ERBB2-CAR CIK cell-treated mice (*n* = 8), whereas mice treated with WT CIK cells only transiently responded, but ultimately progressed in 2 of 6 (33%) cases (Figures 2D,E, WT, *n* = 6 and ERBB2-CAR CIK, *n* = 8). Tumors were macroscopically cleared in all mice treated with ERBB2-CAR CIK cells, while small macroscopic lesions were observed in some of the mice treated with WT CIK cells (Figure 2C). Accordingly, 4 of 6 (67%) animals treated with WT and all 8 of 8 (100%) animals treated with ERBB2-CAR CIK cells were in complete molecular remission as determined by PCR analysis (Figure 2F). In contrast, CD3-positive human immune effector cells were identified in high numbers in the spleen of WT and ERBB2-CAR CIK cell-treated mice and in lower numbers in the liver but were not detectable by IHC in xGVHD-targeted organs, such as the guts and lungs (Figure 2G and Table 1). Whereas, weak signals were detectable by IHC for both cell types, only low amounts of WT CIK cells but high amounts of ERBB2-CAR CIK cells were detected by PCR. These data demonstrate that ERBB2-CAR CIK cells retain target cell specificity *in vivo*, and either exhibit increased tissue migration or improved persistence compared to WT CIK cells (Figure 2G).

**Table 1:** Biodistribution of WT and ErbB2-CAR CIK cells following preemptive treatment.

	**WT CIK (*****n*** **=** **6)**						**ErbB2-CAR CIK (*****n*** **=** **8)**							
Liver	+	+	–	+	–	–	–	–	+	–	–	+	–	–
Lung	–	–	–	–	–	–	–	–	–	–	–	–	–	–
Spleen	++	+	++	+	++	++	n/a	+++	++	+++	+++	–	+	+
Gut	–	–	–	–	–	–	–	–	–	n/a	n/a	–	–	–

*IHC staining of CD3-positive immune effector cells: –, negative; +, low grade; ++, medium; +++, high grade; n/a, not available*.

### WT and ERBB2-CAR CIK Cell Treatment of Established RMS Tumors

**Figure 3:**
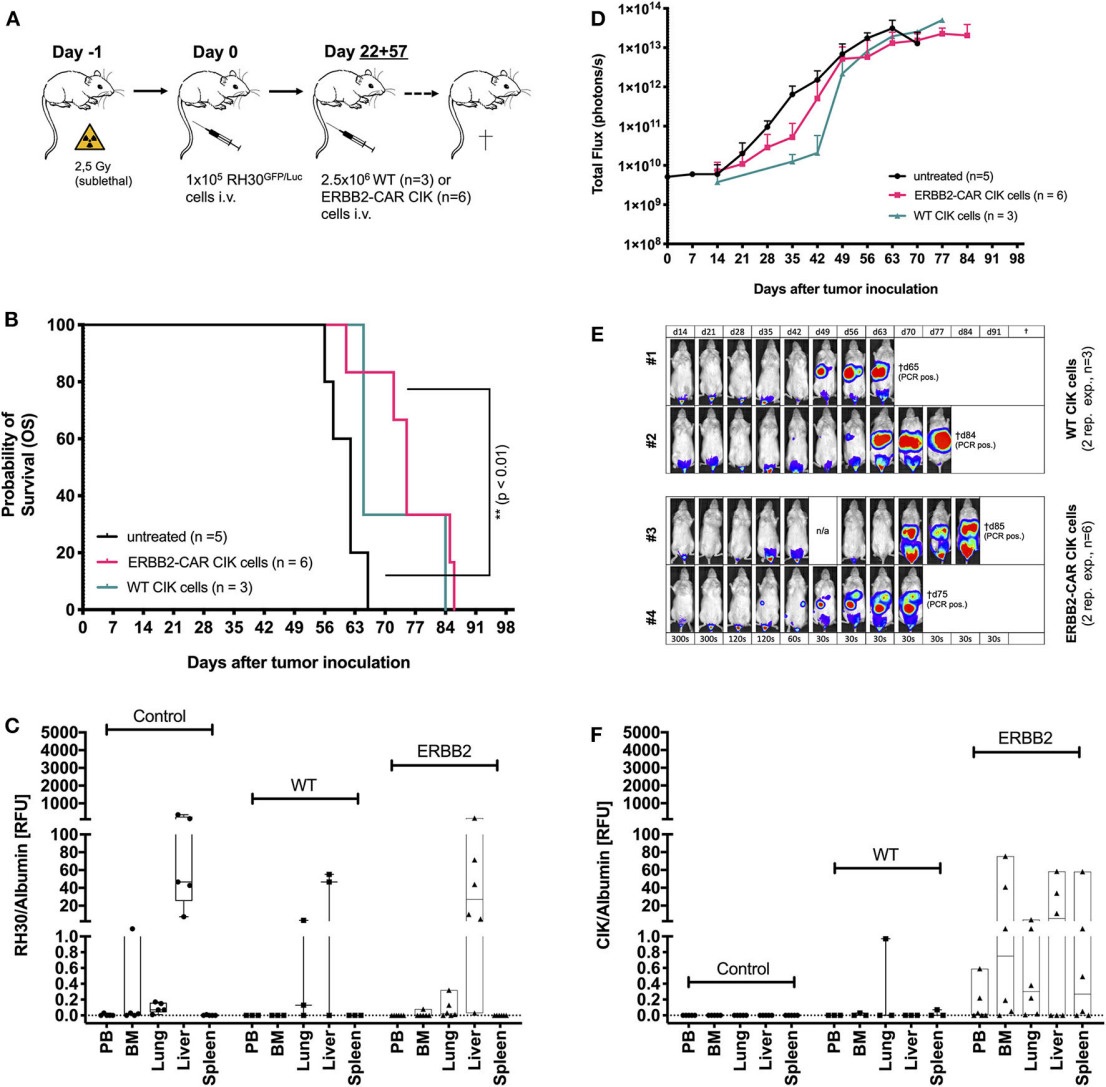
Cytotoxicity of WT and ERBB2-CAR CIK cells against established human RH30^GFP/Luc^ tumors. WT and ERBB2-CAR CIK cell infusions were analyzed for activity against established human RH30^GFP/Luc^ tumors **(A)**. ERBB2-CAR CIK cell treatment significantly improved survival compared to no treatment (*p* < 0.01, **B**). Survival **(B)**, BLI **(D,E)**, and PCR results **(C)** were used to assess tumor engraftment. Of note, ERBB2-CAR CIK cells but not WT CIK cells were detectable by PCR at tumor sites **(F)**.

To model WT and ERBB2-CAR CIK cell therapy during advanced RMS relapse, WT and ERBB2-CAR CIK cell infusions were applied in another group of mice already engrafted with human RH30^GFP/Luc^ tumors (Figure 3A). Mice with delayed ERBB2-CAR CIK cell treatment on days +22 and +57 after tumor cell injection experienced significantly improved survival compared to untreated controls (*p* < 0.01, Figure 3B), whereas WT CIK cell-treated mice only showed a trend toward improved survival (*p* > 0.07). BLI, autopsy, and PCR analysis showed a short delay, but not an abrogation of tumor growth in WT and ERBB2-CAR CIK cell-treated mice compared to untreated controls. Here, antitumor responses were more pronounced in ERBB2-CAR CIK cell-treated mice than in WT CIK cell-treated mice (Figures 3C–E). Of note, ERBB2-CAR CIK cells, but not WT CIK cells were detectable by PCR at tumor sites (Figure 3F), albeit at lower levels compared to preemptive ERBB2-CAR CIK cell therapy (Figure 2G).

### Human ERBB2 Surface Expression of RMS Tumors

At the time of infusion, ERBB2 molecule expression on RH30 cells was identified by flow cytometry. However, expression levels were consistently low, not allowing detection of ERBB2 by standard IHC in all tumor-targeted organs (Figure 1E). Interestingly, IHC analysis of established tumors in spleens confirmed the surface expression of ERBB2 molecules (Figure 1E).

### Biodistribution and Toxicity

**Figure 4:**
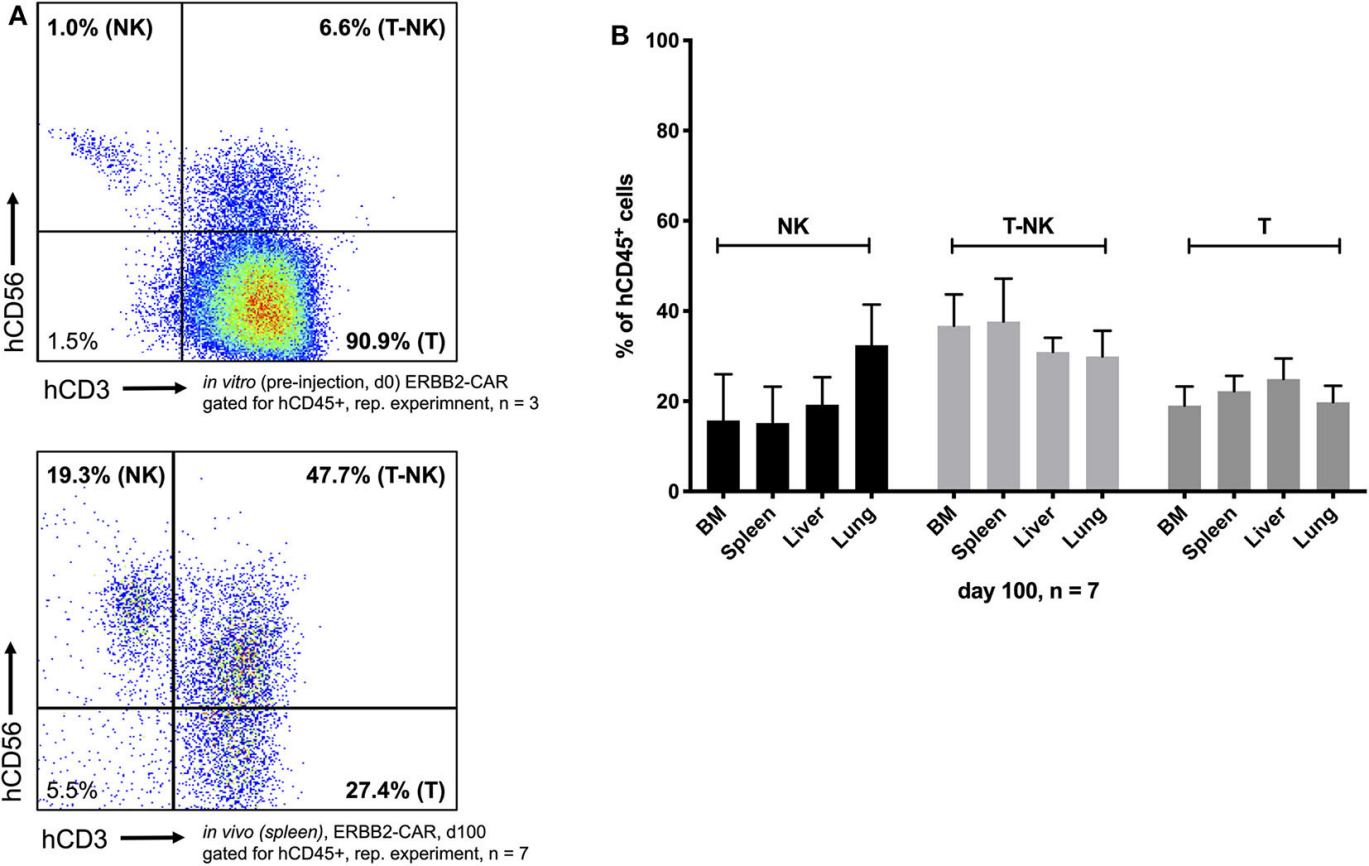
Biodistribution and toxicity model. Two infusions of ERBB2-CAR CIK cells enabled survival of the ERBB2-CAR CIK cells and persistence of innate immune cells within the adoptively transferred heterogeneous CIK cell population, in particular CD3^−^CD56^+^ NK and CD3^+^CD56^+^ T-NK cells (**A** bottom and **B**), which accounted for only 1 and 6.6% of the cells at the time of infusion, respectively (**A** top). ERBB2-CAR and WT CIK cell engraftment took place in all analyzed organs (**B**, Figures 2G, 3F), but did not lead to xGVHD (confirmed by histology, Table 2).

Preemptive ERBB2-CAR CIK cells and, to a lesser extent, WT CIK cells produced an effective antitumor response associated with the presence and prolonged persistence of immune cells at tumor sites. Interestingly, tumor eradication induced by only two infusions of ERBB2-CAR CIK cells enabled survival of the ERBB2-CAR CIK cells, but also led to the expansion and persistence of immune cell subpopulations, namely CD3^+^CD4^+^ helper T cells, CD3^−^CD56^+^ NK cells, and CD3^+^CD56^+^ T-NK cells, which had only been found in very low numbers within the adoptively transferred heterogeneous CIK cell population at the time of infusion (Figures 4A,B, Supplementary Figures 1E,F).

CIK cell engraftment also took place in potential xGVHD-target organs where ERBB2-expressing tumors were present, such as the spleen and lungs. There were no significant differences in numbers or phenotype (Supplementary Figure 1) between WT CIK cells and ERBB2-CAR CIK cells present in the spleen or lungs (Figure 2G). Histopathology of gut and lung tissue of mice injected preemptively with WT or CAR CIK cells showed no infiltration of CD3-positive human lymphocytes and no signs of tissue damage. Lymphocyte infiltration was moderate in livers and was highest in spleens. Histopathological analysis of these organs showed no signs of tissue damage, necrotic hepatocytes, or fibrosis (Tables 1, 2). Furthermore, immune cell engraftment did not lead to severe CRS or xGVHD, but enhanced graft-vs.-tumor effects toward ERBB2-expressing RMS cells (Table 2). Altogether, the infusion of ERBB2-CAR CIK cells was found to be effective, safe, and well-tolerated.

**Table 2:** XGVHD after preemptive treatment with WT and ERBB2-CAR CIK cells.

	**WT CIK (*****n*** **=** **6)**						**ErbB2-CAR CI*****K (n*** **=** **8)**							
Liver	–	–	–	–	–	–	–	–	–	–	–	–	–	–
Lung	–	–	–	–	–	–	–	–	–	–	–	–	–	–
Spleen	–	–	–	–	–	–	–	–	–	–	–	–	–	–
Gut	–	–	–	–	–	–	–	–	–	–	–	–	–	–

*Signs of tissue damage, necrosis or fibrosis assessed by HE staining: –, no signs; +, low signs; ++, medium signs of xGVHD*.

## Discussion

Preclinical studies using CAR T cells from healthy donors have shown that these cells are effective when targeting solid tumors, but this approach is limited when using patient's own T cells for CAR modification (9). In a phase I/II clinical trial, ERBB2-CAR T cells persisted for 6 weeks but ultimately failed to improve the outcome of high-risk sarcoma patients. However, treatment efficacy was improved by lymphodepleting conditioning prior to adaptive transfer of ACT, which further enhanced ERBB2-CAR T cell expansion and persistence *in vivo* (21).

CIK cells—which have documented safety in the autologous and allogeneic setting—may be considered an alternative immune cell source for CAR modification, as patient- and even healthy donor-derived-CIK cells may be used for adoptive immunotherapy of high-risk RMS. Mutations affecting the receptor tyrosine kinase/RAS/PIK3CA pathway, such as mutations in the tyrosine kinase genes *FGFR4, PDGFRA*, and *ERBB2*, are the most common mutations observed in RMS and could be targeted by approved therapeutics (19, 20). Once preclinical *in vivo* safety and efficacy against RMS are demonstrated, ERBB2-CAR CIK cells may enable the rapid preparation of a subsequent planned clinical trial urgently needed for the treatment of high-risk patients with alveolar RMS.

Here, we present an ACT approach targeting CIK cells to ERBB2 with a second-generation CAR for the potential treatment of refractory human alveolar RMS in a completely new xenograft model. In this model, NSG mice carrying small or large tumor burden of RH30^GFP/Luc^ cells that had already spread to BM, liver, lung and spleen at the time of treatment mimic the clinical situation of high-risk patients. The novelty of our study is based on the use of CIK cells rather than T cells targeting ERBB2 *in vivo* in the context of preemptive ACT and during alveolar RMS in its advanced form.

Establishment of human alveolar RMS xenografts was feasible in 100% of mice following tail vein injection of tumor cells. Tumor engraftment was detectable after 13 days by BLI imaging. Samples taken from the PB, BM, lung, liver, and spleen of mice with visible signs of poor health after a median of 62 days (range 56–66 days) reassured human alveolar RMS engraftment. IHC staining confirmed ERBB2 target molecule expression on small tumor lesions but not on established tumors in the liver, probably due to the autofluorescence of the liver tissue and bile.

It is widely recognized that CAR-engineered T cell products lose *in vivo* function during culture, driving the development of ever-shorter manufacturing protocols (43–45). In previous work, we employed CIK cells after *ex vivo* activation with IL-15 for 10 days, which resulted in more rapid generation of CIK cells with stronger cytotoxicity compared to conventional CIK cells expanded in the absence of IL-15 over 3–4 weeks. (32, 46, 47). The main fraction in this short-term, IL-15-activated CIK cell culture is the CD3^+^ T cell population. Modification of IL-15 activated CIK cells by lentiviral CAR transduction and its influence on proliferation, phenotype, anti-tumor ability, cytokine secretion, and alloreactivity *in vitro* was previously published by our group (31). CAR CIK cells efficiently and selectively lysed ERBB2-positive tumor cells, which only showed minimal sensitivity to WT CIK cells, whereas parental ERBB2-negative tumor cells remained unaffected. However, cytotoxicity triggered by interaction of their activating NK receptors with stress ligands expressed by tumor cells was retained by ERBB2-CAR-CIK cells. Target cell recognition triggered secretion of pro-inflammatory cytokines and chemokines such as IFN-γ, TNFα, and MIP-1α/CCL3 as well as release of granzyme B, while production of IL-6 or IL-10—which is associated with CRS—was not observed. Moreover, when co-cultured with freshly isolated PMBCs from HLA-mismatched donors, neither WT CIK nor CAR CIK cells displayed significant alloreactivity *in vitro*. In the actual study, no differences in proliferation nor in the proportions of T, T-NK, and NK cells were found, whereas ERBB2-CAR CIK cells before infusion contained significantly more helper T cells with an effector memory phenotype compared to WT CIK cells which showed more naïve T cells. This may be responsible for the improved overall antitumor capacity of ERBB2-CAR CIK cells compared to WT CIK cells. Mean transduction efficiency determined *via* eGFP expression of transduced CIK cells was 21.1 ± 8.8%, with 84.3 ± 6.9% of these cells also displaying high CAR expression on the cell surface.

Only partial tumor growth inhibition was achieved in WT CIK cell-treated mice, while the *in vivo* antitumor functions of ERBB2-CAR CIK cells resulted in complete elimination of human alveolar RMS in the setting of preemptive therapy. ERBB2-CAR CIK cells showed sustainably migration to distant tumor sites, were capable of penetrating tumor tissues, and showed long-lasting persistence and activity, which we did not observe for WT CIK cells. The transferred ERBB2-CAR CIK cell population was composed of low numbers of NK and T-NK cells and high numbers of T cells at the time of infusion, where at the ERBB2-CAR was exclusively expressed by the T and T-NK cell compartments. Hence, a comprehensive comparison of the *in vivo* antitumor functions of CIK cells to those of young T cells engineered with ERBB2-CAR might be interesting as a means of deciphering whether it is the use of CIK cells that is the basis of the observed activity of this approach. However, the T cells among CIK cells are non-classical terminally differentiated T lymphocytes with an NK cell phenotype representing diverse T and NK cell receptor specificities, which are not comparable to young T cells (31, 48). Furthermore, CIK cells include classical NK cells as well as CD4^+^ and CD8^+^ T lymphocytes with naïve T, effector memory, and central memory T phenotypes, and this may represent an advantage with respect to the unselected CAR-transduced T cell populations used so far, which contain significant amounts of CD4^+^ T cells that may contribute to the dramatic cytokine storm sometimes observed *in vivo* (49). In contrast to the high specificity and memory of CAR T cells, CIK cells are capable of eliminating tumor cells by recognizing pathogen patterns through a variety of receptors (DNAM-1, NKG2D, NKp30, and TCR/CD3) (24), suggesting that ERBB2-CAR CIK cells may provide NK cell-like activities, mainly NKG2D-mediated functions, and specific anti-ERBB2-mediated cytotoxicity in combination, as indicated by NK and T-NK cells which were present at potential tumor sites in all ERBB2-CAR CIK cell-treated mice. However, due to the strong autofluorescence properties of analyzed organs, which interfered with eGFP emission signals, CAR expression was not assessable. Usually, NK and T-NK cells cannot be tracked *in vivo* (50), but infiltrating NK cells and NK cell-mediated antitumor responses have been described to be associated with a relatively good prognosis (51, 52). Here, we showed that multi-specific ERBB2-CAR CIK cell therapy could eliminate low tumor load and that ERBB2-CAR CIK cells could be used as a vehicle for delivering preferentially T_H_-1 cytokines and chemokines directly to tumor sites, which are involved in regulating innate and adaptive immunities and may have allowed T, NK, and T-NK cells to proliferate, persist, and survive *in vivo*, which is in contrast to IL-15 activated WT CIK cells and conventional IL-2-activated CIK cells reported by others (53).

However, ERBB2-CAR CIK cell therapy was not as effective in the treatment of relapsed or progressive disease, even though the immune effector cells reached tumor sites and were increased in numbers compared with WT CIK cells. The tumor microenvironment and tumor cells themselves can shed target molecules, thereby limiting the potential of CAR-modified immune cells when targeting solid tumors (54, 55). Hence, overcoming the general resistance of solid tumors to immunotherapy appears highly warranted. Therefore, ERBB2-CAR CIK cell treatment may be used preferentially in the preemptive therapy setting given in repetitive doses. Furthermore, combination therapies with PD-1/PD-L1 inhibitors or histone deacetylase inhibitors (HDACis) might be considered to increase the antitumor efficacy of ERBB2-CAR CIK cells in patients with resistant alveolar RMS.

Of particular concern in clinical trials is the risk of toxicity. Hence, cytotoxicity is likely to occur even with low levels of the ERBB2 antigen, which may exist on normal tissues, such as those in the respiratory tract. In addition, numerous examples of more severe toxicity occurring with cellular therapy approaches that can kill normal cells with low-level antigen expression have been reported. However, Ahmed et al. previously demonstrated the safety and activity of ERBB2-CAR T cell therapy in 17 patients with sarcoma in a phase I/II clinical trial using a very similar second-generation CAR with the same targeting domain as that employed in our study (9). Due to the non-MHC-restricted NK cell-like cytotoxicity of CIK cells and more importantly, based on the preclinical and clinical observations that CIK cells almost completely lack toxicity, we considered CIK cells as immune effectors for our analyses (28–30). Indeed, our treatment approach was well-tolerated, GVHD-targeted organs of mice showed only minimal infiltration of cytotoxic T lymphocytes, while no signs of tissue damage were observed. However, mice are not a good model for assessing toxicity, as they do not fully recapitulate human toxicity or provide insight into on-target toxicity due to the lack of cross-reactivity between anti-human CARs and homologous murine antigens.

Altogether, in biodistribution and toxicity analyses, ERBB2-CAR CIK cells were safe, well-tolerated, and effective, especially in the model with low tumor burden, which was not likewise observed for WT CIK cells. Thus, CIK cells engineered with lentiviral vectors, with their dual roles as targeted killers and modulators of innate immunity, appear to represent a platform with considerable potential to improve the outcomes of children and young adults with ERBB2-positive high-risk alveolar RMS.

## Data Availability Statement

The raw data supporting the conclusions of this article will be made available by the authors, without undue reservation.

## Ethics Statement

The animal study was reviewed and approved by Regierungspräsidium Darmstadt, Germany Gen.-Nr. TVA FK/1000.

## Author Contributions

MM, JW, WSW, EU, and ER: conceived and designed the experiments. MM, JW, HK, and ER: performed the experiments. MM, HK, CH, LMM, WSW, PB, and ER: analyzed the data. JW, HK, WSW, HB, ZI, and EU: contributed to reagents, materials, and analysis tools. MM and ER: wrote the manuscript. HK, CH, LMM, WSW, HB, ZI, EU, TK, and PB: revised the manuscript. PB and TK: supervised the research. All authors: approved the final version of the manuscript.

## Conflict of Interest

The authors declare that the research was conducted in the absence of any commercial or financial relationships that could be construed as a potential conflict of interest.

## Supplementary Material

Supplementary Figure 1 Phenotypic characterization of the WT and ERBB2-CAR CIK cells *in vitro* and *in vivo*. The development of CIK cell subpopulations during expansion of unmodified CIK cells and CIK cells transduced with the lentiviral CAR vector was investigated by flow cytometric analysis of the surface markers CD3 and CD56 **(A)**, as well as CD4 and CD8 **(A)**, and their memory phenotypes **(C,D)**. Results obtained with CIK cells before infusion are shown, including the rate of CIK cells with EGFP marker expression **(A)**. Cell surface expression of ERBB2-CARs was confirmed using a secondary anti-IgG-Fc monoclonal FACS antibody against an ERBB2-IgG-Fc chimera (**B**), one representative experiment shown, *n* = 3. Additionally, the biodistribution of WT and ERBB2-CAR CIK cells with helper T/CD4^+^ and cytotoxic T/CD8^+^ phenotype at the end of the *in vivo* experiment (preemptive treatment group) was assessed by flow cytometry **(E,F)**.
