# Supplementary figures and images for: ERBB2-CAR-Engineered Cytokine-Induced Killer Cells Exhibit Both CAR-Mediated and Innate Immunity Against High-Risk Rhabdomyosarcoma
Source: Front Immunol. 2020 Oct 19;11:581468. doi: 10.3389/fimmu.2020.581468 (PMC7641627; doi:10.3389/fimmu.2020.581468)

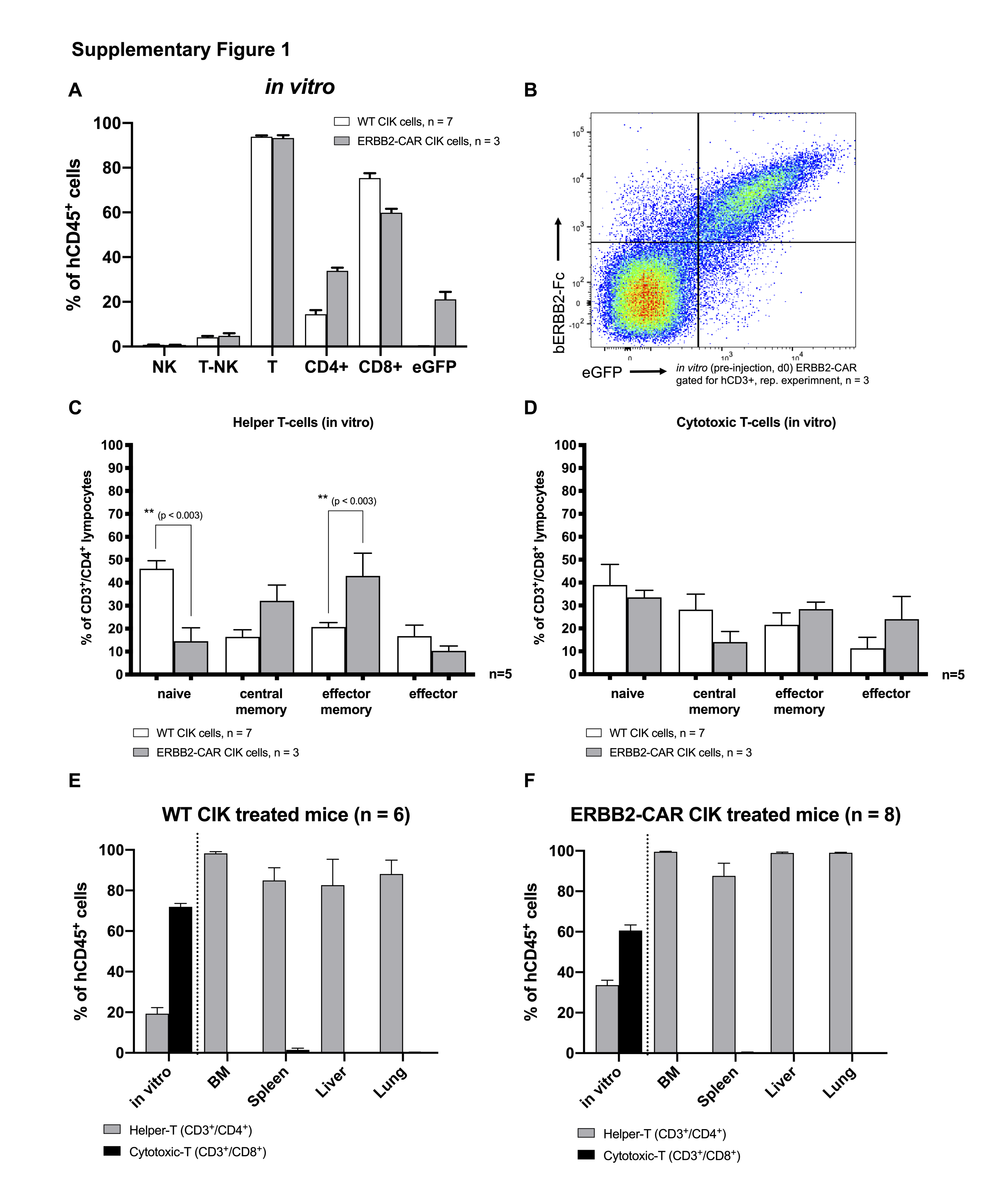

Supplement: Supplementary Figure 1 — Phenotypic characterization of the WT and ERBB2-CAR CIK cells in vitro and in vivo. The development of CIK cell subpopulations during expansion of unmodified CIK cells and CIK cells transduced with the lentiviral CAR vector was investigated by flow cytometric analysis of the surface markers CD3 and CD56 (A), as well as CD4 and CD8 (A), and their memory phenotypes (C,D). Results obtained with CIK cells before infusion are shown, including the rate of CIK cells with EGFP marker expression (A). Cell surface expression of ERBB2-CARs was confirmed using a secondary anti-IgG-Fc monoclonal FACS antibody against an ERBB2-IgG-Fc chimera (B), one representative experiment shown, n = 3. Additionally, the biodistribution of WT and ERBB2-CAR CIK cells with helper T/CD4+ and cytotoxic T/CD8+ phenotype at the end of the in vivo experiment (preemptive treatment group) was assessed by flow cytometry (E,F). [file Image_1.TIFF]
